# Individual differences in crowding predict visual search performance

**DOI:** 10.1167/jov.21.5.29

**Published:** 2021-05-26

**Authors:** Inês S. Veríssimo, Stefanie Hölsken, Christian N. L. Olivers

**Affiliations:** 1 Cognitive Psychology, Institute for Brain and Behavior, Vrije Universiteit Amsterdam, Amsterdam, The Netherlands

**Keywords:** visual search, crowding, functional viewing field (FVF), peripheral vision

## Abstract

Visual search is an integral part of human behavior and has proven important to understanding mechanisms of perception, attention, memory, and oculomotor control. Thus far, the dominant theoretical framework posits that search is mainly limited by covert attentional mechanisms, comprising a central bottleneck in visual processing. A different class of theories seeks the cause in the inherent limitations of peripheral vision, with search being constrained by what is known as the functional viewing field (FVF). One of the major factors limiting peripheral vision, and thus the FVF, is crowding. We adopted an individual differences approach to test the prediction from FVF theories that visual search performance is determined by the efficacy of peripheral vision, in particular crowding. Forty-four participants were assessed with regard to their sensitivity to crowding (as measured by critical spacing) and their search efficiency (as indicated by manual responses and eye movements). This revealed substantial correlations between the two tasks, as stronger susceptibility to crowding was predictive of slower search, more eye movements, and longer fixation durations. Our results support FVF theories in showing that peripheral vision is an important determinant of visual search efficiency.

## Introduction

Trying to find what we are looking for is common to our everyday lives. Moreover, anyone who has looked for an item in a supermarket aisle has experienced that such visual search behavior is limited, as we cannot attend everywhere simultaneously. Hence, central to visual search is the decision on where to look next—a decision that involves mechanisms of perception, attention, and memory (Chan & Hayward, 2013; Eimer, 2015; Nakayama & Martini, 2011; Wolfe, 2015). In modeling such decisions, researchers have focused on the influence of both stimulus properties (e.g., Itti & Koch, 2000), as well as top-down and contextual factors (e.g., Castelhano & Henderson, 2007; Wolfe & Horowitz, 2017).

An important question is what the underlying causes of the limitations in visual search are. The dominant theoretical framework over the past four decades has assumed that central, covert attentional mechanisms make up the main bottleneck in visual search (Wolfe, 2014). This line of theory goes back to at least (Hoffman, 1979) but received a major boost with Treisman's Feature Integration Theory (Treisman & Gelade, 1980), which posited that while target objects defined by single salient features may be detected in parallel, objects that can only be distinguished as a combination of features require covert attention to be directed to them, in a serial fashion. Wolfe's Guided Search model (Wolfe et al., 1989; Wolfe, 1994; Wolfe & Gray, 2007) also assumes that visual search is essentially limited by the speed with which covert attention can be directed to individual items within a single glance, resulting in what has been referred to as the “car wash” model. In this model, items enter a serial processing stream one-by-one, even though multiple items can be “in the process” at a time (up to a maximum of four). Which items are eligible for entering the car wash is then mainly determined by top-down weighting of task-relevant features, which occurs in parallel across the visual field. This architecture of initial parallel stage of feature processing followed by a second stage involving some central attentional bottleneck is still echoed in much of modern thinking about visual search (e.g., Eimer, 2015; Liesefeld & Müller, 2020; Wolfe, 2017).

Note that hitherto, central bottleneck theories have assumed the input to be of homogeneous resolution across the visual field—though see Wolfe (2003) for pointing out that this does not have to be the case. In fact, the parallel feature detection stages of these theories rely on such features being readily available across the visual field. However, the visual system is heavily *foveated*, as already starting in the retina, photoreceptor density rapidly decreases with eccentricity (e.g., Curcio et al., 1990). In addition, compared to the fovea, peripheral vision is assigned much less of the cortical surface, as is expressed in the cortical magnification factor (Duncan & Boynton, 2003). This results in worse detection of visual search targets in the periphery unless stimuli are rescaled according to this factor (Carrasco et al., 1995, 1998; Carrasco & Frieder, 1997; Motter & Simoni, 2007). Moreover, attention itself appears to be biased toward the center of vision (Wolfe et al., 1998), and attentional resolution—defined as the smallest spatial region within which individual objects can be selected by visual attention—is severely limited in the periphery with even a coarser scale than the resolution of the input signal (Intriligator & Cavanagh, 2001; He et al., 1996). Together, many of these factors probably contribute to what is known as *crowding* (Bouma, 1970; Toet & Levi, 1992; Wolford & Chambers, 1983; see Levi, 2008; Whitney & Levi, 2011, for reviews). Crowding is the phenomenon upon which stimuli become difficult to impossible to discriminate when embedded in clutter and occurs through competitive interactions between the target and surrounding stimuli (the flankers). Crowding is most pronounced in peripheral vision, although studies have also shown effects of crowding in central vision (Coates et al., 2018; Lev et al., 2014). It imposes a major limitation on peripheral vision, impairing perception beyond mere acuity (Rosenholtz, 2016). However, none of these limitations play any role in central bottleneck theories of visual search.

This contrasts with a different class of theories that does not assume homogeneous resolution. Instead, these theories see the above mentioned inherent limitations of peripheral vision as the major determinant of visual search efficiency, rather than some central covert attentional bottleneck (Akbas & Eckstein, 2017; Engel, 1977; Geisler & Chou, 1995; Hulleman & Olivers, 2017; Motter & Simoni, 2008; Rosenholtz et al., 2012; Rosenholtz, 2016; Zelinsky, 2008). These theories can be collectively described as FVF theories, after the construct of the “functional viewing field” (FVF; also known as “useful field of view”; Ball et al., 1988; Sanders, 1970). The FVF is the area of the visual field, centered on fixation, within which a certain target stimulus can still be discerned. By definition, the size of the FVF is determined by any factors that limit peripheral vision, as listed above. In contrast to central bottleneck theories, FVF theories of search assume that all stimuli are processed in parallel within one eye fixation. Instead, the seriality of search emerges when the FVF is smaller than the to-be searched area, and thus not all display items can be sufficiently discriminated from peripheral vision. The solution to deal with these limitations is for the system to make eye movements, which bring potential target candidates back within the FVF. In other words, search becomes serial because eye movements are serial and not because of a central cognitive limitation (Hulleman & Olivers, 2017). Several computational implementations have indeed generated positive results in terms of predicting both manual responses and eye movements (Hulleman & Olivers, 2017; Najemnik & Geisler, 2005; Parkhurst et al., 2002; Young & Hulleman, 2013; Zelinsky, 2008). Moreover, Akbas and Eckstein (2017) have shown that a foveated convolutional neural network generates search performance that comes close to a network with full resolution input but at considerably reduced computational (and hence metabolic) costs.

FVF theory predicts that visual search efficiency is determined by the efficacy of peripheral vision, rather than a central bottleneck (Hulleman & Olivers, 2017). The present study tested this prediction by capitalizing on individual differences in sensitivity to crowding. Specifically, we hypothesized that individuals who present stronger crowding should also show less efficient visual search. We focused on crowding as it is such a strong determinant of peripheral vision, and thus the FVF. Previous studies have provided evidence for a potential link between crowding and visual search performance by showing that when the discrimination of target stimuli in the periphery becomes more difficult—be it due to either closer proximity or similarity between targets and nontargets—search for a target under the same circumstances also becomes less efficient (Engel, 1977; Geisler & Chou, 1995; Gheri et al., 2007; Motter & Simoni, 2008; Wertheim et al., 2006). For example, both Wertheim et al. (2006) and Gheri et al. (2007) found that targets defined by a unique and salient feature relative to surrounding distractors can be distinguished better than targets defined by a conjunction of features, both in a peripheral detection task and in a visual search task. This provides initial qualitative evidence that what benefits one task also benefits another. Using a different approach, Rosenholtz and colleagues have argued that a single model of statistical transformations of different stimulus types in peripheral vision can explain both crowding and visual search performance (Rosenholtz et al., 2012; Zhang et al., 2015). They showed that the peripheral discriminability of statistically transformed stimuli indeed predicts search efficiency (with better predictability than classic search models, as can been seen in Chang & Rosenholtz, 2016). However, they did not investigate a direct empirical link between classical measures of crowding and visual search.

**Figure 1.**
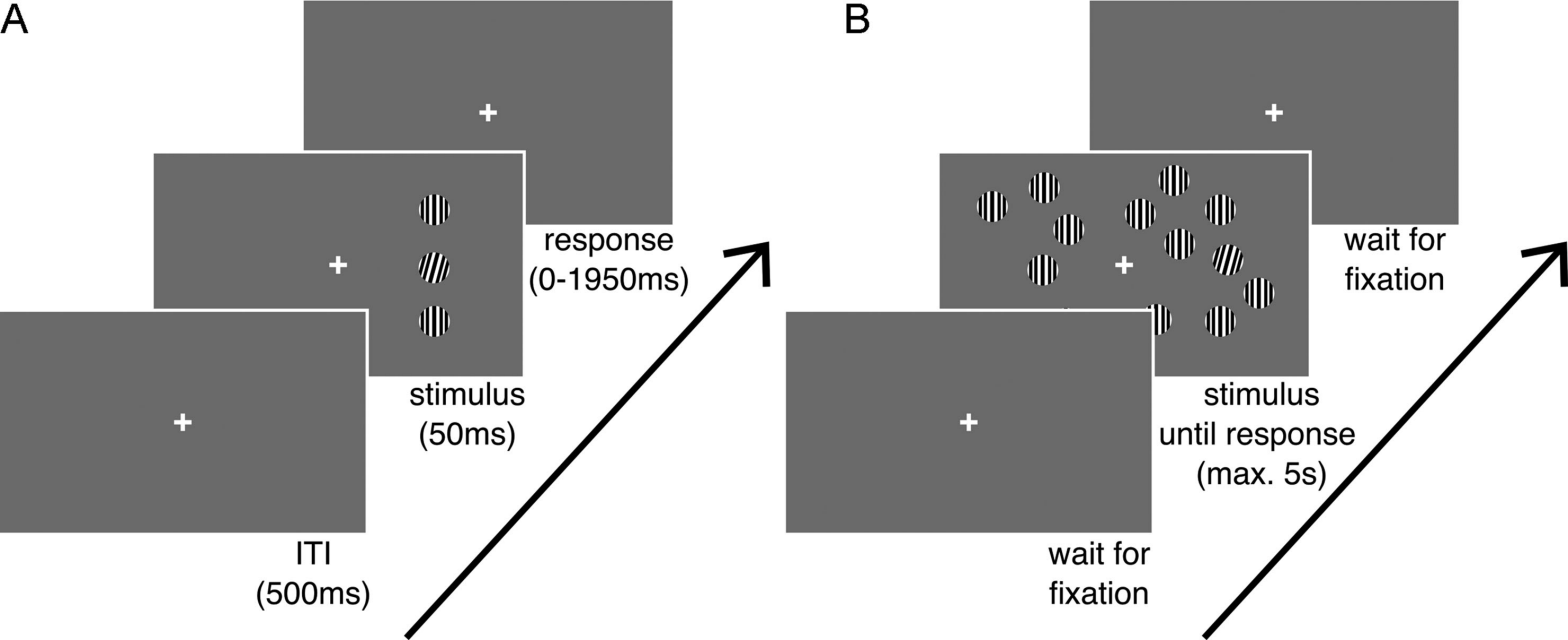
Schematic illustration of stimuli and experimental procedure of both tasks. (A) Crowding task. A tilted target Gabor patch with two flankers was shown for 50 ms, after which participants had up to 1,950 ms to indicate the direction of tilt. Stimuli could appear at one of three possible eccentricities (4, 8, and 12 deg), on either the left or the right side of the fixation mark. Distance between target and flankers was staircased per eccentricity. (B) Visual search task. Displays consisted of a tilted target Gabor patch surrounded by distractors, making a total of 5, 15, or 30 items on screen. The target was presented at the same eccentricities as used for the crowding task. Participants indicated the tilt direction of the target.

Here we aimed to more directly test the link between crowding and search by using a well-established measure of crowding, referred to as the critical spacing (CS). The CS is the minimum distance below which target-flanker proximity impacts discrimination performance (Whitney & Levi, 2011). In his original study, Bouma (1970) estimated the CS to be around half the eccentricity, but it is known to depend on stimulus properties (e.g., Rosen et al., 2014; Scolari et al., 2007) as well as attention (Grubb et al., 2013; Rashal & Yeshurun, 2014; Strasburger & Malania, 2013; Yeshurun & Rashal, 2010). Additionally, studies have shown that crowding magnitude differs across individuals (Frömer et al., 2015; He & Fang, 2019), and it is this variability that we made to use in this study. To this end, we devised an experiment consisting of two parts, both of which are illustrated in Figure 1. In the first part, the crowding task, participants discriminated the orientation of a target Gabor pattern at three fixed eccentricities left or right from fixation. On most trials, the target was accompanied by two flankers. A staircase procedure varying the target-flanker distance allowed us to estimate the CS for each individual, which we defined as the spacing that allowed for ∼80% accuracy. In the second part, the same participants then completed a visual search task, using the same type of stimuli, but now randomly arranged and with larger set sizes. We hypothesized that the individual CS values, as obtained in the first part, would be predictive of search performance both in terms of manual Reaction Times (RTs) as well as eye fixations. A more detailed description of the methods follows below.

## Materials and methods

The study design and analyses were preregistered at the Open Science Framework (https://osf.io/mv8hw/). Customized code used to produce the figures and values presented in this document can be found at https://github.com/iverissimo/Crowding.

### Participants

Participants were recruited from the student population of the Vrije Universiteit Amsterdam. Participants were naïve with respect to the purpose of the experiment and reported normal or corrected-to-normal vision. All participants gave written informed consent and were compensated for their time (either financially or through course credits). The study procedures were approved by the ethics committee of the Faculty of Behavioral and Movement Sciences. Sample size was predetermined using G*Power (Faul et al., 2009), assuming a moderate effect size of 0.6, which indicated a minimum sample size of 26. We decided on a minimum of 30 participants meeting the predetermined inclusion criteria, but allowing for as many additional participants as feasible within the time period of the study (with no optional stopping). In the end, a total of 62 participants were recruited, of whom 44 met the inclusion criteria and were entered in subsequent analyses (7 male, mean age 20, age range 18–31). Inclusion criteria were defined as follows: With regards to the crowding task, accuracy on the target alone condition, without flankers, should not be below 60% (note that accuracy on the trials with flankers was staircased to 75%). Additionally, participants should have no more than 25% of missed trials (trials where no response was given) and should not deviate their gaze from fixation (>1 degree of visual angle — deg) during stimulus presentation for more than 10% of the trials, in any of the conditions. Finally, to prevent floor or ceiling effects, the staircased estimates of CS should not be at minimum or maximum values. After running the experiment, we realized that we obtained three CS values (one for each eccentricity), and we decided to only include participants for whom at least two out of the three CS estimates had not reached bottom or ceiling. With regards to the search task, participants were included if overall accuracy (across set sizes) was at least 85% and at least 75% for any given set size.

### Experimental setup

The session consisted of two tasks: first a crowding task, followed by a visual search task. The entire session lasted about 60 to 90 min, and participants were allowed to take breaks between blocks and tasks. For both tasks, visual stimuli were generated with custom-made code developed with Python 2.7 (Python Software Foundation, Beaverton, OR, USA), using Psychopy functions (Peirce et al., 2019). The display was presented on a Samsung SyncMaster 2233RZ monitor (native resolution of 1,680 × 1,050, screen height 30 cm, and refresh rate of 120 Hz), with a gray background (mean luminance of 15 cd/m${}^{2}$), at a viewing distance of 57 cm. A chinrest was used to stabilize participants’ head position. Eye movements were recorded throughout both parts of the experiment using an EyeLink 1000 remote eye-tracker system (SR Research, Ontario, Canada) with a refresh rate of 1,000 Hz. At the start of each task, a standard calibration-validation procedure was performed.

### Experiment Part I—crowding task

Figure 1A illustrates the procedure of the crowding task. Throughout the experiment, a white cross of 0.5 deg diameter was positioned at the center of the screen, and participants were asked to keep fixation on that point at all times. Each trial displayed a target Gabor patch on either the left or the right side of the fixation cross, and subjects were asked to indicate its tilt (7 deg from vertical, either clockwise or anti-clockwise) by pressing the right or left arrow keys. This target Gabor could appear either on its own (one sixth of the trials) or accompanied by two vertically positioned flanker patches (five sixths of the trials), with vertical orientation. To avoid grouping by collinearity, a slight jitter was applied to the flanker positions by randomly shifting them horizontally between −0.5 and 0.5 deg relative to the target. All presented Gabor patches had a diameter of 2.2 deg and a spatial frequency of 4 cycles/deg (standard deviation of 0.12 deg, Michelson contrast of 99.5%). The stimuli were displayed for 50 ms at an eccentricity of 4, 8, or 12 deg from central fixation, after which participants had an additional 1,950 ms to respond. The intertrial interval (ITI) was 500 ms, during which only the fixation mark was presented. The crowding task was divided into four equal-length blocks, with a total of 576 trials. The experiment was preceded by an additional 72 practice trials. Eccentricity, visual hemifield (left/right), and presence of flankers were balanced and presented in a randomized order. The distance between target and flankers was staircased according to participants’ performance in a 1-up-3-down scheme, with a fixed step size of 0.05 deg. At the beginning of the task, the distance between target and flankers was set at the maximum of 0.80 × eccentricity, and this value decreased if the participant correctly indicated (in three consecutive trials) the orientation of the target, up to a minimum value of 0.20 × eccentricity. Three separate staircases were used, one per eccentricity level.

### Experiment Part II—visual search task

Figure 1B illustrates the visual search procedure. Fixation cross, background, and Gabor patches were the same as in the crowding task. Search displays comprised a 22-deg (wide) by 14-deg (tall) virtual oval grid of 156 possible item positions, arranged in 11 concentric rings, with a minimum distance of 2 deg between adjacent items. Item position was randomized per trial. After participants directed their gaze at the fixation mark, a search display—composed of a tilted Gabor target patch among vertically oriented distractor patches—appeared. Participants were instructed to find the target as quickly as possible and indicate the target orientation by pressing the right or left arrow keys. In contrast to the crowding task, observers were now free to move their eyes. The search display disappeared after response, with only the fixation cross remaining. A new trial would start once participants returned their gaze to fixation. The experimental design contained two main factors. First, display set size was varied between 5, 15, and 30 items. Second, the target was presented at an eccentricity of 4, 8, or 12 deg from central fixation. All levels were balanced and randomly mixed within blocks. There were four equal-length blocks of 180 trials each, giving a total of 720 trials, and 80 trials per set size × eccentricity combination.

### Data processing and analyses

Behavioral and eye movement data from both tasks were analyzed with custom-written code in Python 3.6. All following processing and analyses steps here were preregistered. Any unforeseen or additional, more explorative analyses will be pointed out in the Results section.

*Crowding data.* Average response accuracy with and without flankers was compared using a Wilcoxon signed-rank test (because of the expected non-Gaussian distribution of the data). The main measure of interest was the CS, which was estimated for each participant per eccentricity. The CS was computed as the median target-flanker over eccentricity ratio of the last 96 trials (the last one sixth of the total number of trials). These ratios were then averaged over eccentricities, to produce one mean CS value per participant.

*Visual search, manual response data.* For the RT analysis, we excluded incorrect trials, and RTs faster than 250 ms or slower than 5,000 ms after stimulus onset. Mean RTs were then computed per participant, for each combination of set size and eccentricity, and submitted to a two-way repeated-measures analysis of variance (ANOVA) with set size and eccentricity as factors, and α=0.05. As a secondary measure, we also computed average response accuracy and entered this in the same type of ANOVA. Next, we fitted a linear regression to the individual RT data in order to compute the slopes of the search functions across set size, for each target eccentricity.

*Visual search, eye-tracking data.* The criteria for fixation and saccade detection were based on the Eyelink standard criteria. Eye-tracking data were processed using the PyGazeAnalyser package (Dalmaijer et al., 2014). We excluded early fixations (up to 150 ms after display onsets) as well as target fixations, since these were not deemed indicative of the search process itself. For each participant, we then computed the average number of fixations, per set size and eccentricity, and submitted these to the same ANOVA as above. We also computed slopes across set size.

*Correlation analyses.* Crucially, to assess if crowding performance was indeed predictive of visual search performance, we then correlated the CS obtained in the crowding task with the abovementioned visual search measures. Specifically, we correlated CS with the search RTs for each set size and eccentricity, the RT × set size slopes, the number of fixations for each set size and eccentricity, and the fixations × set size slopes. As these correlations were all planned, were clearly predicted by our hypotheses, and were all predicted to be interrelated (and thus to go in the same direction), they were not Bonferroni corrected. However, as we had no a priori assumptions about the nature of the relation (whether linear or not) and normality of the distributions of the underlying measures, we chose the more conservative option of the Spearman correlation (a nonparametric measure of rank correlation) over the Pearson correlation.

## Results

### Crowding

Table 1 shows the average accuracy for flanker and no-flanker trials, for each eccentricity. As would be expected, accuracy on trials with flankers was significantly worse than on trials without flankers (83% vs. 89%; $p=6.64\times 10^{-6}<0.001$; this also held for each eccentricity separately, all with p<0.01). Note that, due to the staircase, the actual values here have limited meaning, but the result indicates that the flankers caused crowding. Note further that performance on the no-flanker trials was well above chance and did not significantly vary with eccentricity (Friedman test with p=0.203>0.05; see also Supplementary Figure S1), suggesting overall little limitation in terms of visual acuity.

**Table 1.** Average accuracy for trials with and without flankers, for each eccentricity, as well as averaged across eccentricity.

	4 deg	8 deg	12 deg	Average
Flankers	81.3%	83.8%	82.7%	82.6%
No flankers	91.3%	88.8%	86.6%	88.9%

**Figure 2.**
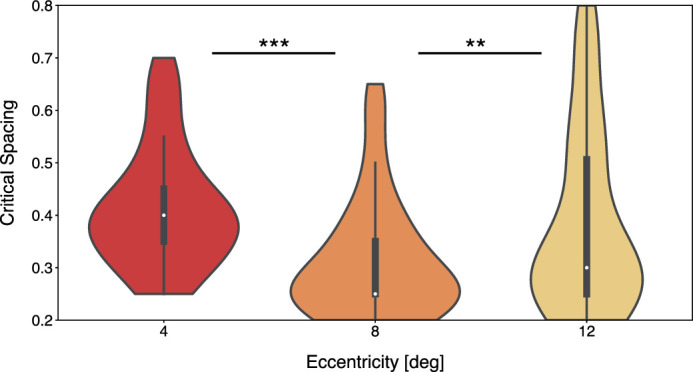
Violin plots of the average critical spacing values and their distribution, per eccentricity. Asterisks indicate the significance levels for the pairwise comparisons (**p<0.01; ***p<0.001).

Figure 2 shows the average CS and its distribution for each eccentricity. Given Bouma's law, we expected the CS values to remain relatively constant across eccentricity (Bouma, 1970). However, a Friedman test revealed a significant effect of eccentricity ($p=2.09\times 10^{-7}<0.001$). Figure 2 indicates that CS was lower for the middle than for the inner and outer eccentricity difference, which was confirmed with post hoc Bonferroni-corrected Wilcoxon tests of the pairwise comparisons (4 vs. 8 deg, $p=2.37\times 10^{-6}<0.001$; 8 vs. 12 deg, $p=4.18\times 10^{-4}<0.01$; with no significant difference between 4 and 12 deg, p=0.07). We did not expect this relative benefit for the middle eccentricity, and we suspect this may reflect a strategy of attending to the center of the eccentricity distribution. Given that this is not crucial for the current purpose, and given a high correlation of CS values between eccentricities, we took the average CS value across eccentricities per participant as the main indicator of crowding for the correlation with visual search performance here. In addition, we report all analyses for each eccentricity-specific CS estimate separately in the Supplementary Figures.

### Visual search

**Figure 3.**
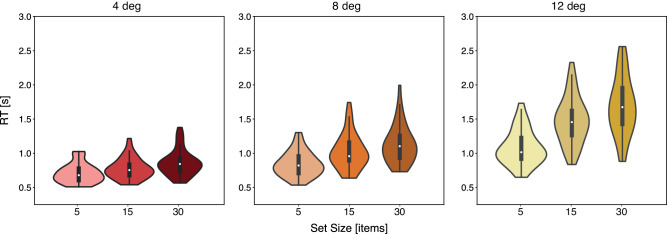
Violin plots of mean search RTs and their distributions, as a function of set size and eccentricity.

Figure 3 shows the mean search RTs as a function of set size, separately for each target eccentricity. A two-way repeated-measures ANOVA with set size (5, 15, and 30 items) and eccentricity (4, 8, and 12 deg) as factors revealed significant effects of set size, $F\left(2,86\right)=202.70,p=2.83\times 10^{-33}<0.001$; eccentricity, $F\left(2,86\right)=298.99,p=1.89\times 10^{-39}<0.001$; as well as their interaction, $F\left(4,172\right)=88.79,p=8.68\times 10^{-41}<0.001$. As can be seen from Figure 3, search times increased with set size and with targets being further away from the center, and these effects amplified each other.

**Figure 4.**
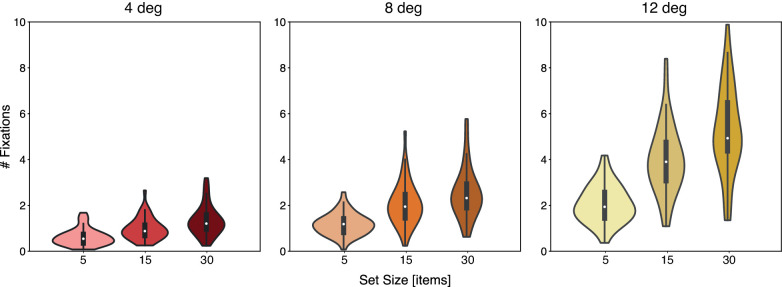
Violin plots of mean number of fixations in the search task and its distribution, as a function of set size and eccentricity.

The same analyses were performed on the number of fixations. Figure 4 shows the distribution of the average number of fixations as a function of set size and eccentricity. The ANOVA revealed a significant main effect of set size, $F\left(2,86\right)=196.47,p=8.54\times 10^{-33}<0.001$; eccentricity, $F\left(2,86\right)=277.50,p=3.08\times 10^{-38}<0.001$; as well as an interaction, $F\left(4,172\right)=119.63,p=1.32\times 10^{-48}<0.001$. Similar to the search times, number of fixations too increased with increasing eccentricity and set size, and these effects amplified each other.

Finally, Table 2 shows the average search accuracy values per set size and target eccentricity. The same ANOVA on these values revealed a significant main effect of set size, $F\left(2,86\right)=14.82,p=2.94\times 10^{-6}<0.001$; eccentricity, $F\left(2,86\right)=61.30,p=2.84\times 10^{-17}<0.001$; as well as an interaction between them, $F\left(4,172\right)=10.18,p=2.03\times 10^{-7}<0.001$. Overall accuracy was high, and the pattern followed that of the RTs, with diminished accuracy for both increasing eccentricity and set size. There was therefore no sign of a speed/accuracy trade-off.

**Table 2.** Average accuracy in the search task, per set size and eccentricity.

	4 deg	8 deg	12 deg
5 items	97.8%	97.8%	95.8%
15 items	97.9%	96.4%	94.2%
30 items	97.7%	96.2%	91.3%

### Correlation analyses

**Figure 5.**
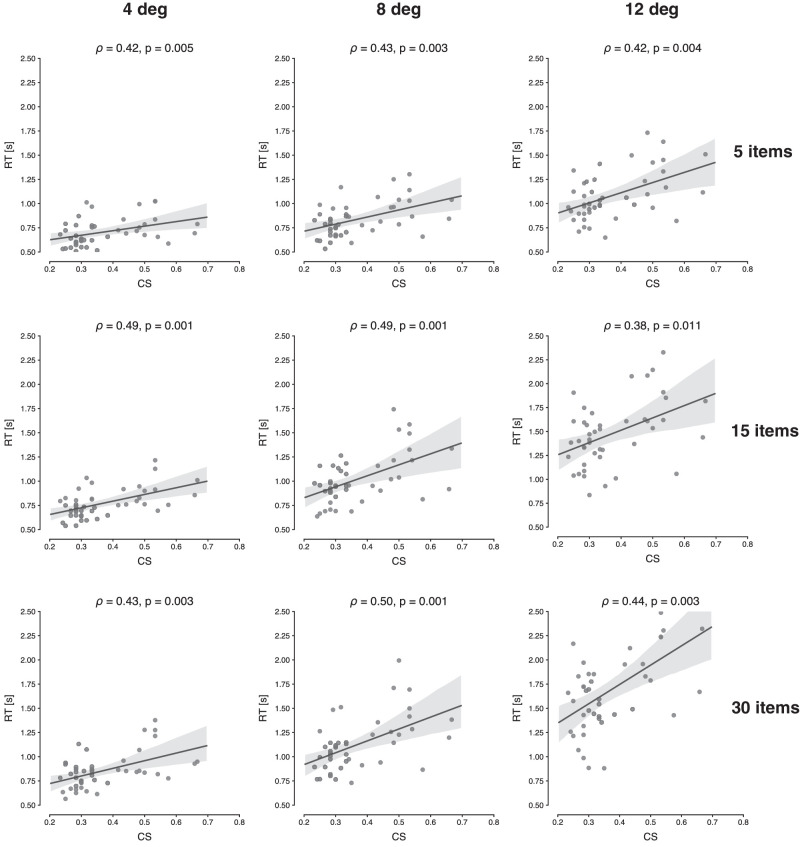
Scatterplots showing the relationship between mean CS and search times, for each set size and eccentricity. The computed Spearman correlation coefficient (ρ) and associated *p*-value are shown at the top of each panel.

To investigate if the CS values obtained from the crowding task are indeed predictive of visual search performance, we computed the Spearman correlation coefficient between crowding and several search outcomes. Figure 5 shows the correlations between CS and search RT, per eccentricity and set size. We can observe that, for all conditions, the correlations are positive and statistically significant (ρ between 0.38 and 0.50, ps≤0.01). Supplementary Figure S2 presents the same analyses, but then taking the CS value for each eccentricity separately. It shows essentially the same results.

**Figure 6.**
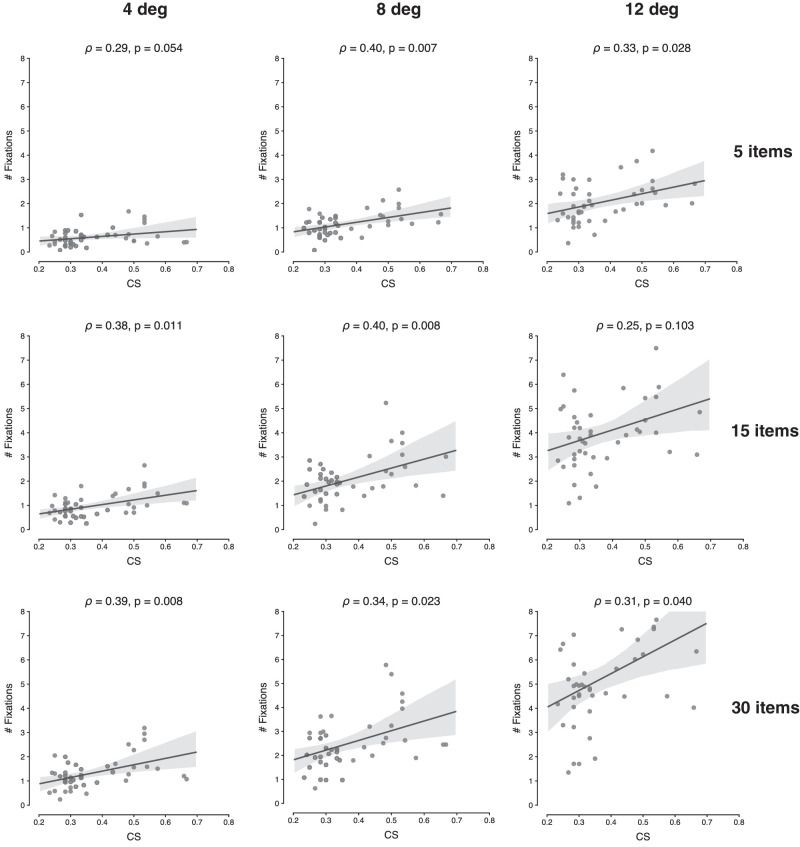
Scatterplots showing the relationship between mean CS and number of fixations performed during search, for each set size and eccentricity. The computed Spearman correlation coefficient (ρ) and associated *p*-value are shown at the top of each panel.

Analogous results were found when correlating CS with the number of fixations that participants made during the search task. In Figure 6, we can observe that for the majority of conditions, the correlations are significant and positive, with ρ ranging between 0.31 and 0.40, ps<0.05, with the exceptions of set size 5, eccentricity 4 deg (ρ=0.29,p=0.054), and set size 15, eccentricity 12 deg (ρ=0.25,p=0.103). Supplementary Figure S3 presents the same analyses, but then taking the CS value for each eccentricity separately. It again shows essentially the same results.

**Figure 7.**
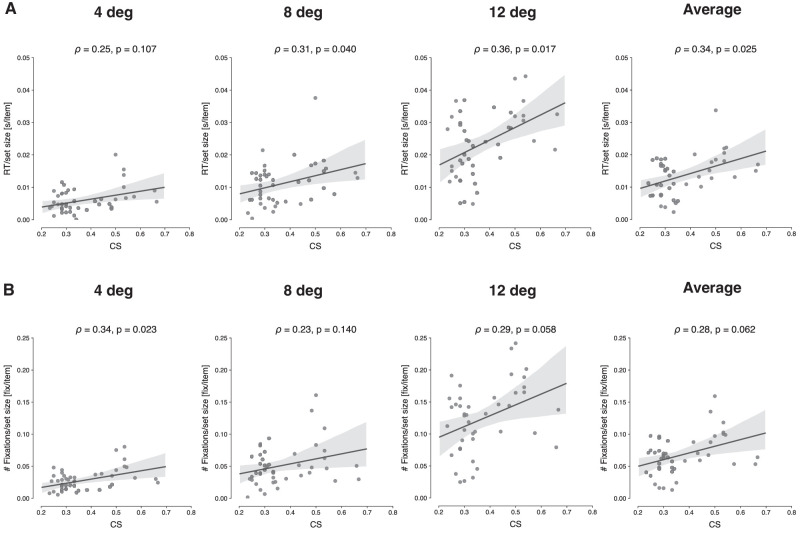
Scatterplots showing the relationship between mean CS and search slopes, for each eccentricity separately and for all combined. The computed Spearman correlation coefficient (ρ) and associated *p*-value are shown at the top of each panel. (A) Average RT/set size slopes. (B) Average fixations/set size slopes.

Figure 7A shows the correlation between CS values and search efficiency—specifically the search slopes across set size as computed using simple linear regression. Slope values showed a significant correlation with CS for search trials where the target was presented at 8 deg (ρ=0.31,p=0.040<0.05), as well as 12 deg (ρ=0.36,p=0.017<0.05), and there was a nonsignificant trend in the same direction at 4 deg (ρ=0.25,p=0.107). The correlation was reliable when aggregated across eccentricities (ρ=0.34,p=0.025<0.05). Figure 7B shows same the correlations, but now for the number of fixations slope values. From the results, we observe that the overall pattern follows a similar trend as for the RT data, yet with somewhat reduced reliability, as the positive correlation only reached significance at 4 deg (ρ=0.34,p=0.023<0.05). Supplementary Figure S4 presents the same analyses, but then taking the CS value for each eccentricity separately. Here results show a strong correlation at 12 deg, but correlations are less reliable for the remaining eccentricities. The overall pattern is again similar, though.

As another measure of peripheral vision, we also correlated search RTs and number of fixations with accuracy in the no-flanker condition. These correlations are shown in Supplementary Figures S5 and S6. Here we used the separate scores for each eccentricity, as in contrast to CS, acuity would be expected to be worse for items further away. As can be seen, there is little correlation with acuity for the lower eccentricities. However, for the largest eccentricity, search performance did correlate with accuracy. This may reflect acuity limitations for targets presented at the furthest eccentricity, even when presented alone. However, note that average discrimination performance was actually not reliably worse for this eccentricity than for the other eccentricities (see Table 1), and performance was well above chance. This indicates that observers could well discern the target tilt even at the furthest eccentricity, making an acuity explanation somewhat unlikely. We will return to this in the Discussion.

### Exploratory analyses

**Figure 8.**
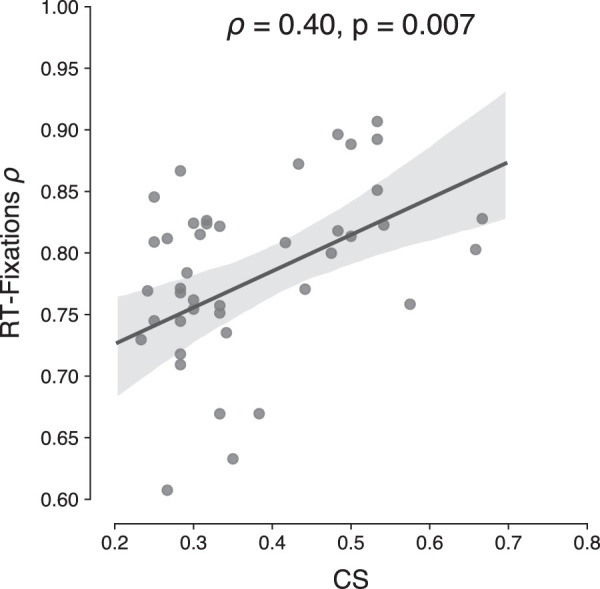
Scatterplot showing the relationship between mean CS and the RT—number of fixations correlations across trials, for each individual separately. The computed Spearman correlation coefficient (ρ) and associated *p*-value are shown at the top of each panel.

We conducted a number of additional analyses. First, FVF theories predict a strong correlation between number of eye movements and RTs. Also, given the strong correlation of both RTs and fixations with CS values here, one would expect RTs and fixations to correlate. To assess this, we computed the RT—number of fixations correlation across trials, for each individual separately. This yielded a strong correlation, with an average ρ=0.78 (*SD* = 0.08; range 0.55–0.91) across participants. We in turn correlated these correlations with the CS values from the crowding task, under the assumption that especially for those individuals who are most limited in their peripheral vision, RTs would be most strongly determined by the number of eye movements. Indeed, as Figure 8 shows, we found a positive correlation between CS and the extent to which an individual's RTs are driven by eye movements (ρ=0.40,p=0.007<0.01).

**Figure 9.**
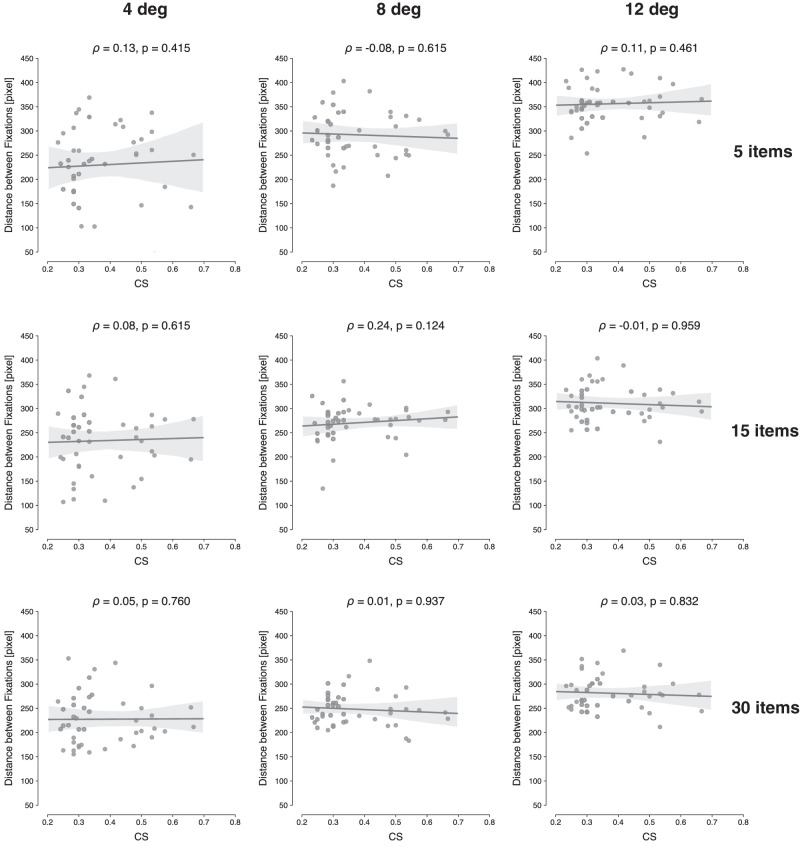
Scatterplots showing the relationship between mean CS and mean distance between consecutive fixations in the search task, for each set size and eccentricity. The computed Spearman correlation coefficient (ρ) and associated *p*-value are shown at the top of each panel.

**Figure 10.**
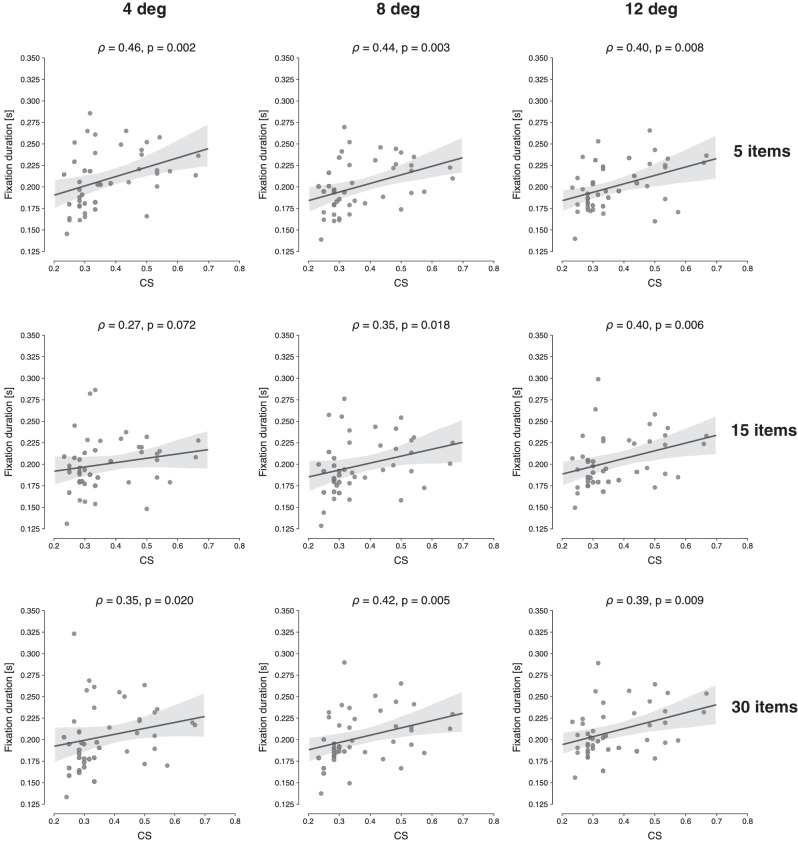
Scatterplots showing the relationship between mean CS and mean fixation duration in the search task, for each set size and eccentricity. The computed Spearman correlation coefficient (ρ) and associated *p*-value are shown at the top of each panel.

Additionally, we were interested in analyzing two other eye movement parameters during search and how these would link to the sensitivity to crowding. In these exploratory analyses, we first computed the average distance between consecutive fixations, for each condition, and correlated these distances with the mean CS. We assumed that participants with higher CS values would have a shorter distance between fixations, since the limits on peripheral vision should lead to smaller FVFs. However, as can be seen in Figure 9, there was no correlation between mean CS and interfixation distance (ρ between −0.08 and 0.24, all p>0.05). Second, we analyzed the average fixation duration and correlated this measure with the mean CS values, as show in Figure 10. Here the assumption was that observers may compensate for worse peripheral vision by fixating longer (cf. Moffitt, 1980). Results show that participants with a larger CS indeed fixated on items longer during search. For the majority of conditions, the correlations are positive and significant, with ρ ranging between 0.35 and 0.46, all p<0.05, with the exception of set size 15, eccentricity 4 deg (ρ=0.27,p=0.072).

## Discussion

Theories of visual search differ with respect to the importance they attribute to central, cognitive versus more peripheral, sensory limitations in explaining search efficiency. Here, leveraging individual differences, we investigated if a major limitation on peripheral vision, crowding, predicts visual search performance. Crowding impairs the discriminability of the object features when surrounded by clutter, and we quantified it by determining the CS between the target object and the flanking objects. We found the CS to vary across individuals, between 0.23 and 0.67, with an average of 0.37, which is not far from the 0.5 proposed by Bouma (1970), with any differences potentially caused by different stimulus properties (Pelli et al., 2007; Rosen et al., 2014; Scolari et al., 2007). However, we did not find the CS to be entirely constant across eccentricity, as the middle eccentricity unexpectedly yielded lower CS values than the inner and outer eccentricities. We believe that this might be an attentional effect, where observers center their attention on the middle of the eccentricity distribution (Eriksen & James, 1986; Juola et al., 1991; Linnell & Humphreys, 2004; Müller & Hübner, 2002), and future investigations might shed more light on this. Important for the present study, the fact that the CS varied across individuals is consistent with earlier findings on individual differences in crowding (Frömer et al., 2015; He & Fang, 2019), and enabled us to link performance to visual search.

The visual search task revealed effects of both eccentricity and set size on manual RTs and accuracy, as well as number of eye movements. Previous studies have shown similar eccentricity effects (Carrasco et al., 1995; Scialfa & Joffe, 1998), which could be the outcome of decreased resolution (Carrasco & Frieder, 1997; Carrasco et al., 1998; Motter & Simoni, 2007) or a by-product of a central bias in the allocation of attention (Wolfe et al., 1998). We point out that while these earlier studies show that targets are more difficult to find the larger the eccentricity, they did not test whether differences in peripheral vision (other than eccentricity per se) are a determinant of visual search performance—which was the goal of the current study. Note that here, in our study, the eccentricity was defined relative to the initial central fixation and would thus change with subsequent eye movements, but averaged across fixations this would still result in an eccentricity difference. Also note that while set size effects were reliable, they were small, with average RT/set size slopes ranging from around 6 ms/item for the nearest target eccentricity to around 23 ms/item for the furthest, suggesting that a considerable part of search may have been parallel in nature (Treisman & Gelade, 1980). We point out that such parallel search reduces the opportunity to find evidence for the core predictions of FVF theory, namely, a direct relationship between search RTs and eye movements, and a relationship between peripheral perception (specifically crowding here) and search. We therefore believe that the fact that we nevertheless find substantial evidence for such relationships (as we will discuss next) is testimony to the power of our approach.

FVF theory predicts that discrimination capacity in peripheral vision is what determines visual search performance (Hulleman & Olivers, 2017). A number of findings support this prediction. First, subjects with higher sensitivity to crowding (i.e., higher CS values), also produced longer RTs during the search task. This strong positive correlation between CS and RTs was observed for all eccentricity levels and set sizes. The same pattern was also observed when correlating search efficiency, as expressed in RT/set size slopes, with CS, although with a more modest effect. This might be due to the fact that overall efficiency was already quite high, and stronger correlations may be observed for less efficient searches. These results are consistent with earlier findings that coupled peripheral discriminability with search efficacy, as expressed in manual responses (Gheri et al., 2007; Rosenholtz et al., 2012; Sayim et al., 2011; Vlaskamp & Hooge, 2006; Wertheim et al., 2006; Zhang et al., 2015). While these studies explored the effects of variations in target-distractor similarity manipulations and found that what is easily discriminable in one task is so too in another, here, by directly correlating a classic measure of crowding with typical search performance outcomes, we provide another important bridge between crowding and visual search.

Furthermore, the same positive correlations were observed between CS and number of fixations, showing that higher sensitivity to crowding leads individuals to make more eye movements during search. When correlating efficiency (here the number of fixations over set size) with CS, we again observed that the overall pattern follows a similar trend as for the RT data, yet with somewhat reduced reliability. Not surprisingly, then, also strong overall correlations were found between the number of eye movements and manual RTs across trials, in line with previous reports (Zelinsky & Sheinberg, 1995, 1997). Moreover, these individual RT-fixation correlation values themselves correlated with the individual CS values, indicating that those individuals who are most limited in their peripheral vision RTs also rely most strongly on the number of eye movements. In support of FVF theories, our results thus show that the spatiotemporal dynamics of visual search can be directly linked to the neurophysiological differences between central and peripheral vision—that is, in overt vision—without having to assume a covert central bottleneck. When an observer's FVF does not encompass all the objects in the display, he or she resorts to eye movements as a serial solution, thus bringing the target object within the FVF.

In light of these findings, we then explored whether varying degrees of crowding leads to different eye movement strategies. Specifically, we speculated that if the FVF is small—leading to more eye movements—then maybe these movements would also show smaller amplitudes, reflecting a denser sampling of the search array. However, no correlation was found between CS and distance between consecutive fixations. This suggests that observers sampled more of the display (as expressed in more fixations), but not in a very systematic fashion (i.e., not in smaller steps). Instead, there was a positive correlation between CS and average duration of fixation. These results are akin to previous studies (Jacobs, 1986; Moffitt, 1980; Motter & Simoni, 2008; Zelinsky & Sheinberg, 1995) and suggest that the FVF may be best conceived of as a dynamic construct, namely, the area of the visual field within which a target stimulus can be discriminated *within a certain amount of time*—something Geisler and Chou (1995) have referred to as the “speed window”. That is, the FVF not only reflects whether sufficient evidence is eventually accumulated in order to detect a target but also the accumulation rate. Longer fixations then compensate for slower evidence accumulation.

While individual differences in crowding were predictive of visual search performance, they explained far from all the variability. This may reflect inherent noise in the stimulus displays, the measurements, or behavior. But it also leaves room for additional factors determining search efficiency, such as systematic scan paths (Gilchrist & Harvey, 2006), biases toward clusters or center of gravity of multiple items (Najemnik & Geisler, 2005; Pomplun et al., 2003), and even covert attentional factors (Carrasco & McElree, 2001; Carrasco & Yeshurun, 1998; Giordano et al., 2009; Wolfe & Gray, 2007). So it deserves pointing out that while the current findings provide positive evidence for FVF theories, they do not provide evidence against central bottleneck theories. That said, and following earlier proponents (Carrasco et al., 1995; Engel, 1977; Eriksen & Schultz, 1977; Geisler & Chou, 1995; Hulleman & Olivers, 2017; Rosenholtz et al., 2012), we argue that it is unnecessary to assume central bottlenecks where peripheral bottlenecks may already be doing the job.

What are the key components that lead to the variability in FVF size and thus to less efficient search performance? One explanation for such individual differences might start at the retina. As is well known, the retina is characterized by a systematic decrease in photoreceptor density with eccentricity. Moreover, this decline varies considerably across individuals (Curcio et al., 1990). Correlations between the accuracy of the target-only condition at the largest eccentricity of the crowding task, on the one hand, and search RTs as well as number of fixations, on the other, suggest that limitations at the retinal level could already be predictive of search performance (at least for the furthest eccentricity). However, when analyzing the average discrimination performance in the target-only condition, we observed no reliable difference in accuracy between eccentricities and well above chance performance. This makes acuity per se an unlikely cause. Perhaps then, the important factor is again processing speed, in that observers feel that they cannot resolve the fringes of their FVF *in time* and thus decide to make another eye movement. Another potential source of individual differences lies at the cortical level (which in turn may or may not find its source in retinal differences). A recent neuroimaging study showed that crowding performance related to the size of population receptive fields (pRFs), specifically in V2, where larger pRF sizes came with stronger crowding (He & Fang, 2019). Receptive field overlap is thought to be a major cause of crowding, leading to mutual suppression of signals, pooling, or both (Levi, 2008). Important within the present context, given that pRF size can predict the magnitude of crowding across individual subjects, the distinct possibility arises that pRF size will also predict visual search performance. Finally, there may be individual differences in feedback projections that shape the FVF, for example, through covert attention. Several studies have shown that manipulating attention on search and acuity tasks has effects that are most pronounced at peripheral locations (Carrasco & Yeshurun, 1998; Carrasco et al., 2002; Grubb et al., 2013; Yeshurun & Carrasco, 1999; for a review, see Anton-Erxleben & Carrasco, 2013). Although to our knowledge, it has never been tested, it is likely that the strength of such attentional modulations differs across individuals, thus contributing to the size and flexibility of the FVF. Future work will be needed to test these possibilities.

## Conclusion

To conclude, our results show that individual differences in crowding, as measured by critical spacing, are predictive of visual search performance. The strong relationship between the two tasks demonstrates the relevance of peripheral sensory limits for visual search, without the need to assume more complex, central attention mechanisms.

## Supplementary Material

Supplement 1

Supplement 2

Supplement 3

Supplement 4

Supplement 5

Supplement 6
