# Supplementary material for: Individual differences in crowding predict visual search performance
Source: J Vis. 2021 May 26;21(5):29. doi: 10.1167/jov.21.5.29 (PMC8164367; doi:10.1167/jov.21.5.29)
Supplement: Supplement 1 [file jovi-21-5-29_s001.pdf]

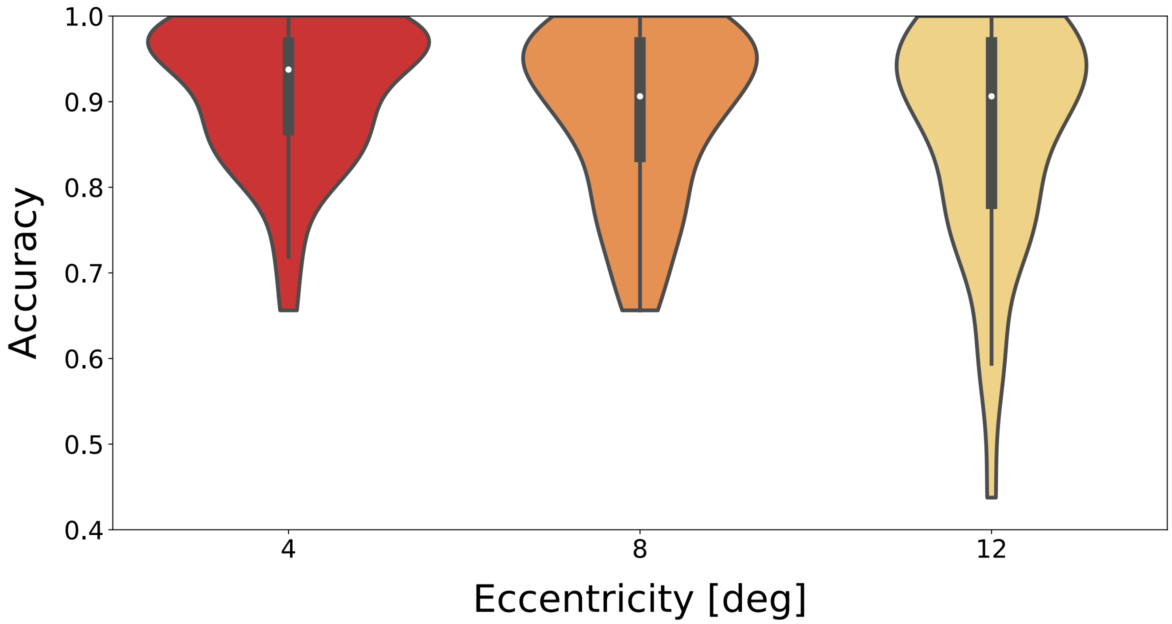

Supplementary Figure 1: Violin plots of the average accuracy values and distribution, for trials without flankers, per eccentricity.
