# Supplementary material for: Individual differences in crowding predict visual search performance
Source: J Vis. 2021 May 26;21(5):29. doi: 10.1167/jov.21.5.29 (PMC8164367; doi:10.1167/jov.21.5.29)
Supplement: Supplement 3 [file jovi-21-5-29_s003.pdf]

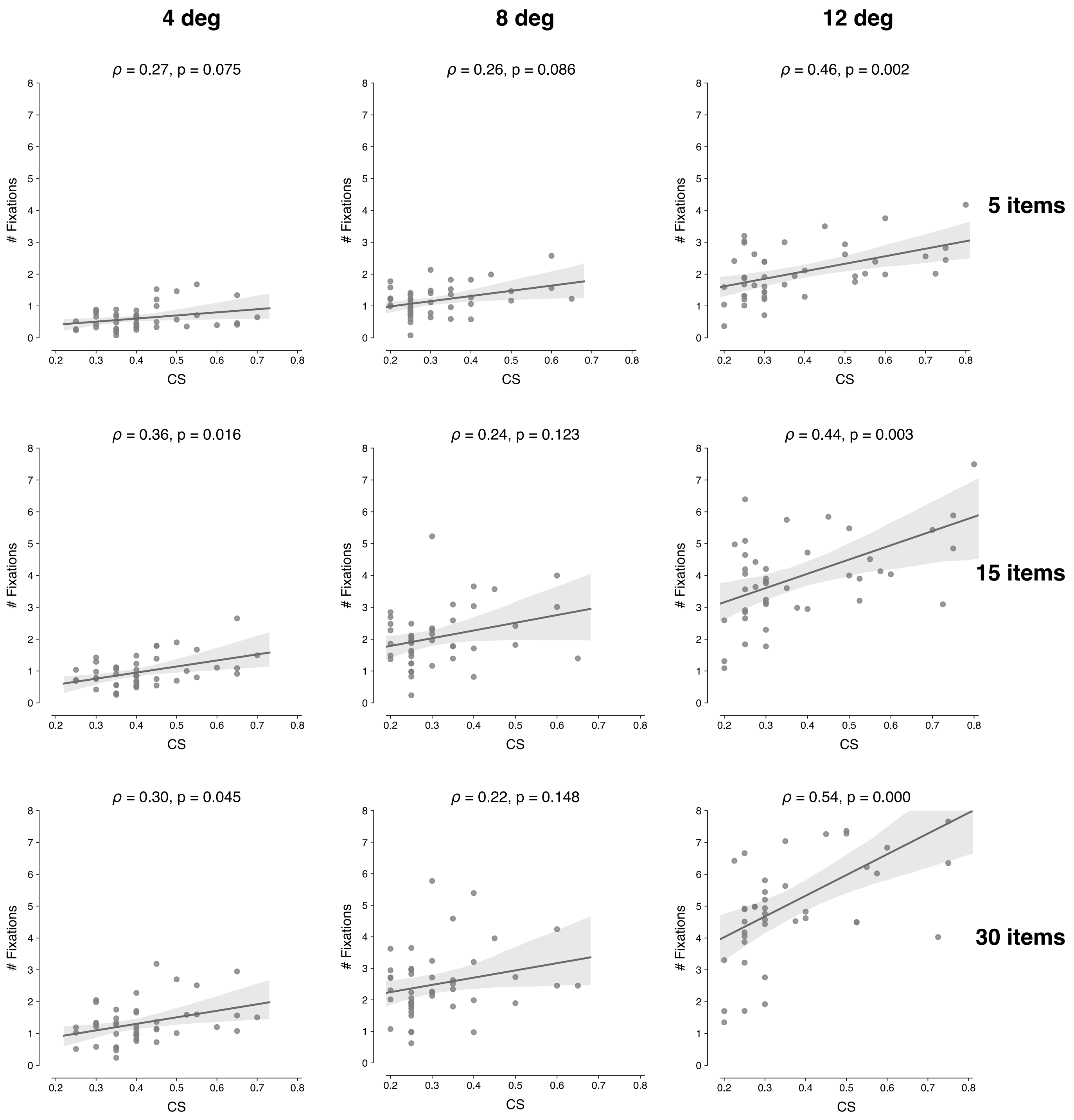

Supplementary Figure 3: Scatter plots showing the relationship between CS and number of fixations performed during search, for each set size and eccentricity. The computed Spearman correlation coefficient (ρ) and associated p-value are shown at the top of each panel.
