# Supplementary material for: Individual differences in crowding predict visual search performance
Source: J Vis. 2021 May 26;21(5):29. doi: 10.1167/jov.21.5.29 (PMC8164367; doi:10.1167/jov.21.5.29)
Supplement: Supplement 6 [file jovi-21-5-29_s006.pdf]

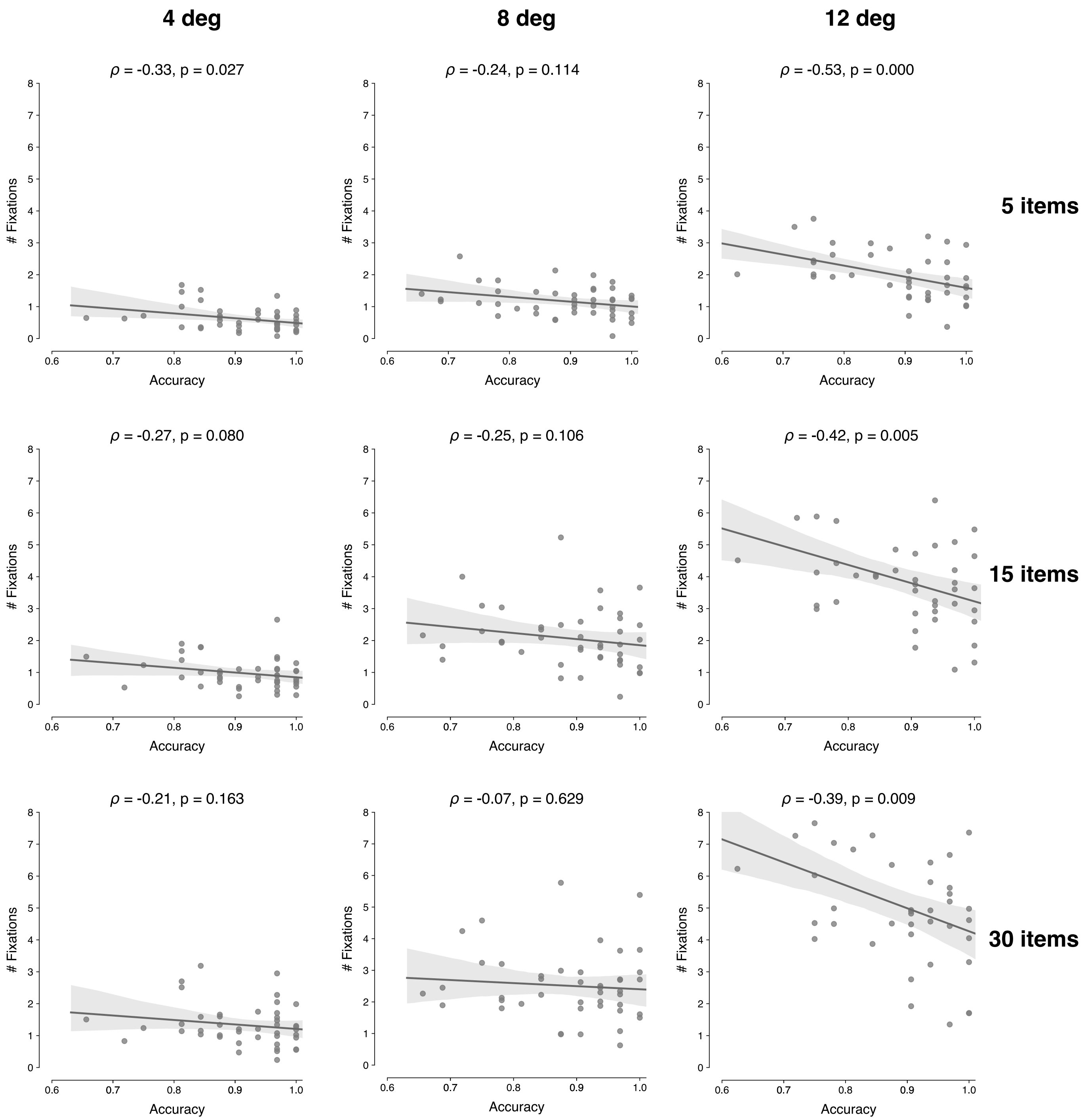

Supplementary Figure 6: Scatter plots showing the relationship between accuracy in the no flanker condition and number of fixations performed during search, for each set size and eccentricity. The computed Spearman correlation coefficient (ρ) and associated p-value are shown at the top of each panel.
